# Photoreceptor Spectral Sensitivity in the Bumblebee, *Bombus impatiens* (Hymenoptera: Apidae)

**DOI:** 10.1371/journal.pone.0012049

**Published:** 2010-08-10

**Authors:** Peter Skorupski, Lars Chittka

**Affiliations:** Biological and Experimental Psychology Group, School of Biological and Chemical Sciences, Queen Mary University of London, London, United Kingdom; Centre de Recherches su la Cognition Animale - Centre National de la Recherche Scientifique and Université Paul Sabatier, France

## Abstract

The bumblebee *Bombus impatiens* is increasingly used as a model in comparative studies of colour vision, or in behavioural studies relying on perceptual discrimination of colour. However, full spectral sensitivity data on the photoreceptor inputs underlying colour vision are not available for *B. impatiens*. Since most known bee species are trichromatic, with photoreceptor spectral sensitivity peaks in the UV, blue and green regions of the spectrum, data from a related species, where spectral sensitivity measurements have been made, are often applied to *B impatiens*. Nevertheless, species differences in spectral tuning of equivalent photoreceptor classes may result in peaks that differ by several nm, which may have small but significant effects on colour discrimination ability. We therefore used intracellular recording to measure photoreceptor spectral sensitivity in *B. impatiens*. Spectral peaks were estimated at 347, 424 and 539 nm for UV, blue and green receptors, respectively, suggesting that this species is a UV-blue-green trichromat. Photoreceptor spectral sensitivity peaks are similar to previous measurements from *Bombus terrestris*, although there is a significant difference in the peak sensitivity of the blue receptor, which is shifted in the short wave direction by 12–13 nm in *B. impatiens* compared to *B. terrestris*.

## Introduction

Bees have long been important models in sensory and behavioural ecology. Among the animals' sensory capacities, much attention has been focussed on colour vision, both as a model for trichromatic and opponent colour processing [1], [2] and as a means of studying learning and generalization abilities [3]. Recently, the North American bumblebee *Bombus impatiens* has become increasingly common as a model organism in such studies [4], and (importantly) in physiological studies of colour processing in the central bee brain [5], [6]. However, detailed spectral sensitivity data are not yet available for this species. A study of bumblebee spectral sensitivity using an optical method includes data from a single *Bombus impatiens* worker, suggesting the presence of blue and UV receptors with peaks close to 450 and 350 nm, respectively [7]. Nevertheless, the photoreceptor inputs underlying colour vision have not yet been measured in this species; instead available data from related species have been assumed to be valid [4], [8].

This assumption may in some circumstances be fairly reasonable, since the Hymenoptera are rather conservative in their colour vision, mostly having UV, blue and green receptors with maximum sensitivities near 340, 430, and 535 nm [9], [10]. Nevertheless species differences do exist. For example, the solitary bee *Callonychium petuniae* has a 4^th^ spectral class of receptor, which appears to be based on a photopigment with a peak sensitivity of about 600 nm [10]. If *Bombus impatiens* is to fulfil its full potential as a model, accurate photoreceptor sensitivity data will be required. We have therefore used intracellular recordings to determine photoreceptor spectral sensitivities in this species.

## Materials and Methods

### Preparation and recording

Worker bumblebees used in these experiments were obtained from commercially available colonies of *Bombus impatiens* (supplied by Biobest Bees, Leamington, Canada). The preparation, and recording and stimulating techniques, were the same as described previously for *Bombus terrestris* [11]. Briefly, a cold-anaesthetised worker was mounted in sticky wax with its head positioned at the centre of rotation of a Cardan arm perimeter device. A small incision was made near the dorsal rim of the right eye for microelectrode insertion. Electrodes were filled with 2 M potassium chloride and had resistances of 80–140 MΩ measured in the retina. The reference electrode was a chlorided silver wire inserted into the contralateral eye. Light was delivered from a 300 W tungsten-halogen lamp via a monochromator (M300, Bentham, UK). The monochromatic beam, whose output was controlled by a set of neutral density filters to vary light intensity over 4 log units, was then focused onto one end of a liquid light guide (3 mm core diameter), the other end of which was mounted on the perimeter device. Once the electrode was in the retina, its approximate position in the eye was ascertained by rotating the Cardan arm to maximize the field response (ERG) to flashes of white or green light, confirming that the optics of the eye were essentially intact and the retina in good physiological condition. The preparation was then left in complete darkness for at least 30 min. On commencement of a recording session, exposure of the preparation to light was restricted by minimizing flash duration and intensity during the search for a photoreceptor. Once stable recordings were obtained, the light source was centred over the photoreceptor's optical axis using the Cardan arm. The opening of the light guide was 6 cm from the eye, subtending a visual angle of 2.9°. Stimulus duration was controlled using a Uniblitz LS2 shutter (Vincent Associates, NY, USA) with a 0.3 ms rise time, and was usually set at 10 ms. The system was calibrated by measuring irradiance with a spectrophotometer (Avaspec–2048; Avantes, Eerbek, NL) using a calibrated UV-Vis light source (DH 2000-CAL, Ocean Optics, Dunedin, Florida). Energy spectra were converted to quantum flux spectra and are expressed in relative log units.

### Measurement and analysis

We used the flash method for measuring spectral sensitivity [12]. First, we determined the intensity-response function at a fixed wavelength by measuring peak depolarization in response to 10 ms flashes of increasing intensity (over 3–4 log units). These data were fitted with a hyperbolic function of the form
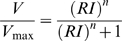
(1)where *V* is the response amplitude (in mV) to a flash of intensity *I* (in quantal flux), *V*_max_ is the saturated response amplitude, *R* is the reciprocal of the intensity giving a response of 50% saturation, and *n* is a constant determining the slope of the function [12], [13], [14]. The spectral sensitivity of the cell would, in principle, be described by repeating this procedure for all wavelengths and determining the variation of the sensitivity parameter *R* with wavelength. In practice this is not possible, and measuring the *V*/log *I* function even over a limited number of wavelengths is time-consuming. Instead we measured the *V*/log *I* function at a single wavelength, and then measured responses to flashes of a single intensity at wavelengths ranging from 320–620 nm in 10 nm increments. The stimuli were not therefore isoquantal, due to the spectral output of the lamp, but we used neutral density filters to restrict response amplitude to the middle, approximately linear region of the *V*/log *I* function. The spectral sensitivity function was then estimated by ‘sliding’ the *V*/log *I* function along the wavelength axis to fit the measured response and then solving for *R* at each measured wavelength . This method assumes that the intensity-response functions at different wavelengths are parallel, as required by the principle of univariance [13].

The resulting spectral sensitivity data were then fitted with simple exponential templates of the form described by Stavenga et al. [15] to estimate the main sensitivity peak (*λ*_max_ ). Where *x* = log(*λ*/*λ*_max_), the sensitivity *S* is described by
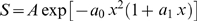
(2)This provided an estimate of *λ*_max_ for each cell, and also provided a means of scaling for pooling of data from different photoreceptors (of the same spectral class) without the distortion that would be introduced by normalizing to one of the fixed 10 nm wavelength increments. In order to describe the complete sensitivity spectrum, we assume that all absorbance bands of the visual pigment are described by the same modified log-normal template [15] of the form
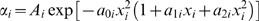
(3)where *i* represents the alpha-, beta- … etc absorbance bands of the visual pigment, and *x_i_* = log(*λ*/*λ*_max *i*_), with *λ*_max *i*_ the peak wavelength for each absorbance band. The complete visual pigment spectrum is then
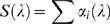
(4)We then fitted our averaged, normalized spectral data with equations (3,4) using the coefficient values tabulated by Stavenga et al. [15] for an A1-based visual pigment; namely, for the alpha band, *A* = 1, *a*_0_ = 380, *a*_1_ = 6.09, with *λ*_max_ as a free parameter, and for the beta band, *A* = 0.29, *a*_0_ = 263, *a*_1_ = 4.45, and *λ*_max_ = 340. We also allowed the tabulated coefficients to vary (within limits) as free parameters in some curve-fitting sessions; see Results for details. Where mean values are given in the text, errors are quoted as one standard deviation.

## Results

Stable intracellular recordings of duration 30–180 minutes were obtained from 33 *Bombus impatiens* photoreceptors. Initial spectral characterization of a cell was done by using the monochromator to swiftly scan the unattenuated output of the lamp across the near-UV, blue, green and orange regions of the spectrum (wavelengths close to 350, 450, 550, and 600 nm at relative log intensities of about 0.03, 0.26, 0.85, and 0.83, respectively). Photoreceptors could quickly be classified as belonging to one of three spectral classes on this basis, as reported previously for *Bombus terrestris* photoreceptors [11]. A given cell generated its largest response at wavelengths close to either 350 (UV), 450 (blue), or 550 nm (green); no cells generated larger responses at 600 nm compared with shorter wavelengths. Further spectral analysis was done on 20 of these cells, confirming that these were UV, blue and green photoreceptors typical of other trichromatic hymenopteran species [10], [11].

**Figure 1:**
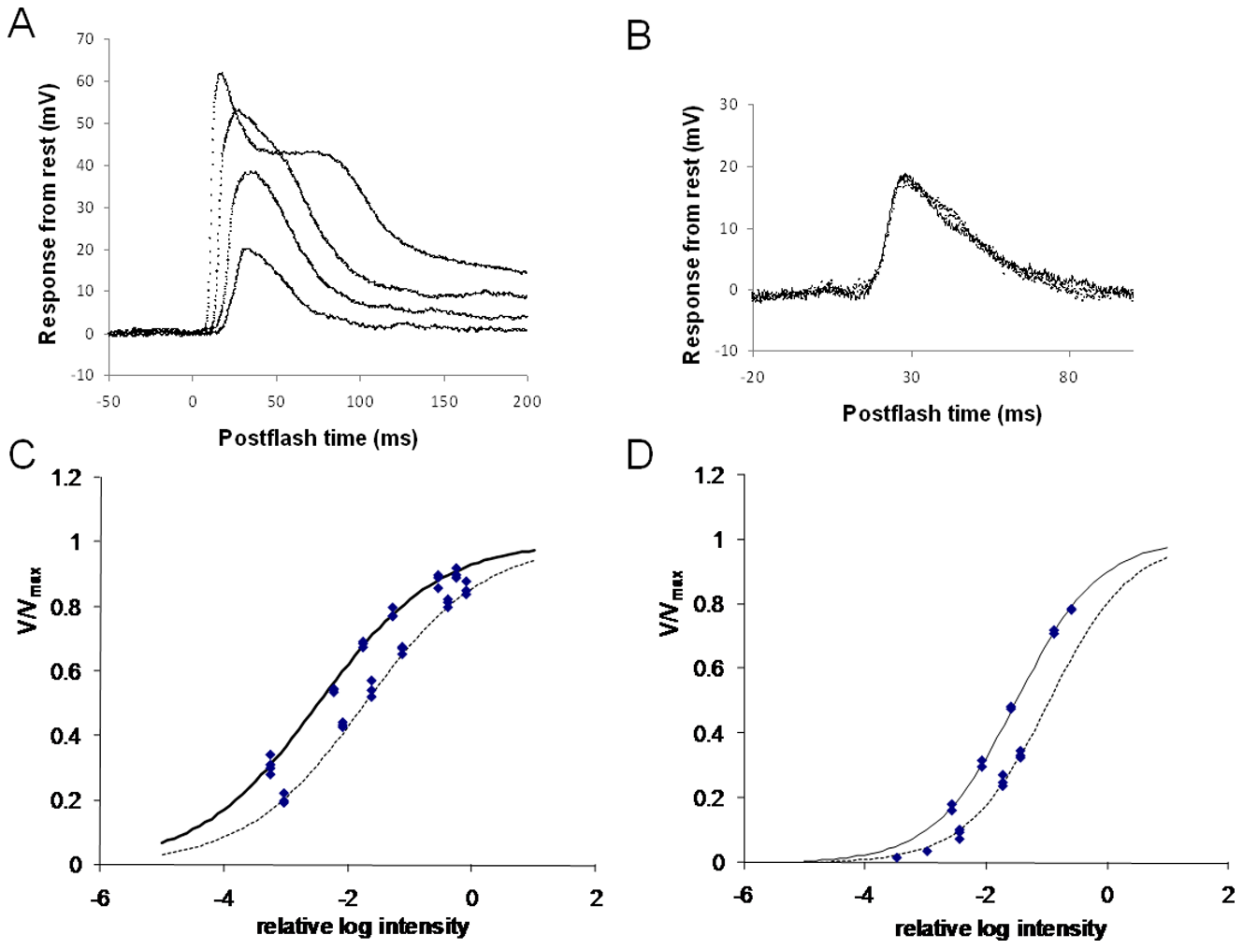
Intracellular recording of photoreceptor responses. A. Superimposed responses of green photoreceptor to 10 ms flashes (onset at time 0) at 550 nm increasing in intensity over approximately 3 log units. B. Superimposed responses from same photoreceptor to flashes at 500 nm (−3.3 log units) and 620 nm (−2.2 log units). C. *V*/log *I* function for a green photoreceptor plotted from responses to 10 ms flashes of varying intensity at 500 and 600 nm. Curves are fits of equation 1 to the data at 500 nm (solid) and 600 nm (dashed). D. Similar data for a blue photoreceptor at 450 nm (solid curve) and 360 nm (dashed curve).

Following initial characterization, a cell was dark-adapted for 3–10 minutes; then its intensity-response function (*V*/log*I* function) was determined at a wavelength close to peak sensitivity. These data were well fitted with the self-shunting equation (equation 1; Fig. 1). Maximal response amplitudes (*V*_max_ ) were extrapolated to be 56.8±5.0 mV for green photoreceptors (n = 9), 50.5±4.7 for blue (n = 5), and 54.2±9.6 for UV (n = 6). In most cases the *V*/log*I* function was measured at a single wavelength. However, in cases where it was measured at a second wavelength, a good fit could be obtained by sliding the function along the intensity axis, so that the estimated values for *V*_max_ and the slope parameter *n*, obtained from fitting the data at one wavelength, could also be used to obtain a reliable fit at a second wavelength, by changing only the sensitivity parameter *R* (as expected from the principle of univariance; Fig. 1C,D).

Low intensity flashes generate responses of somewhat variable latency and amplitude. As intensity is increased, a larger, somewhat noisy, longer duration response is elicited. As intensity is increased still further, rise time and time to peak decrease as response amplitude increases. At the highest intensities a rapid, partial repolarisation of the response peak is evident, so that the response waveform displays an initial spike-like transient followed by a prolonged depolarizing plateau (Fig. 1A). As we varied the stimulating wavelength no departures from the principle of univariance were evident, in so far as responses of similar amplitude generated by different wavelengths (at different intensities) were of essentially the same waveform (Fig. 1B), as would be expected from the principle of univariance, where response depends only on quantal absorption.

**Figure 2:**
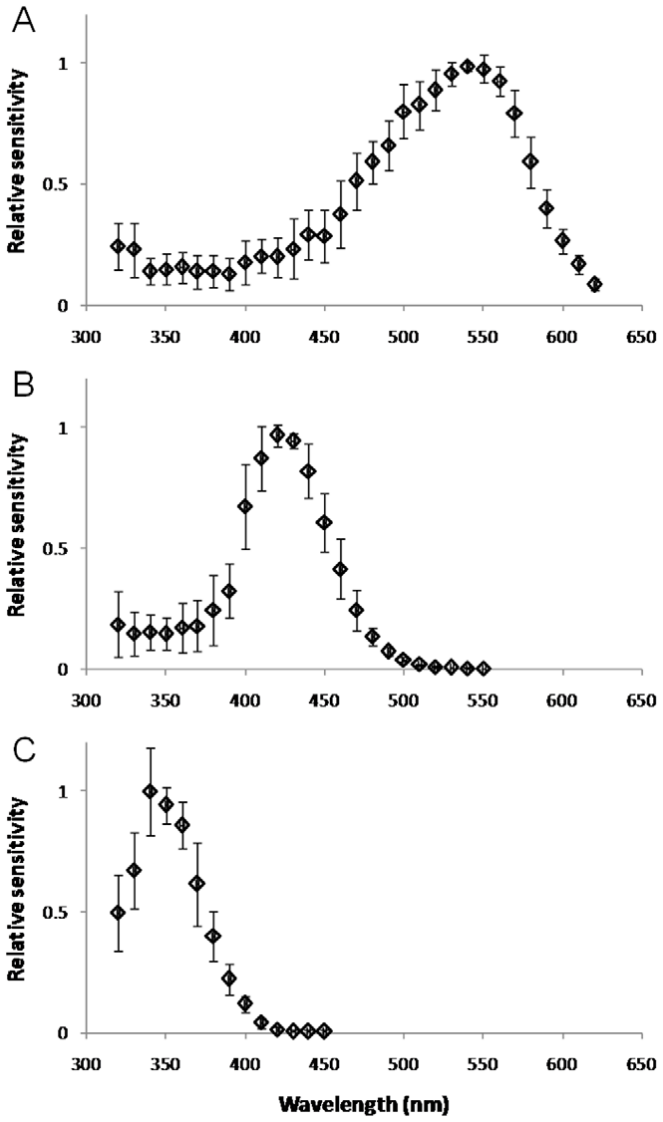
Photoreceptor spectral sensitivity functions. Normalized, averaged data for green (A; n = 9), blue (B; n = 5) and UV (C; n = 6) sensitive photoreceptors. Error bars are ±1.0 SD.

**Figure 3:**
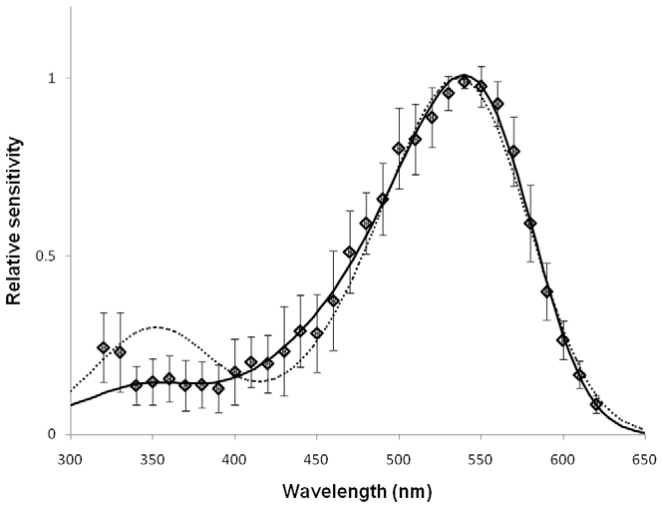
Normalized, averaged spectral sensitivity data from *Bombus impatiens* green photoreceptors (symbols, ±1.0 SD) fitted with equations (3,4). Dotted curve: best fit using tabulated coefficients from [15], yielding λ_max_ = 536 nm. Solid curve: fit with same equations, but allowing coefficients to vary as free parameters, converging as follows: λ_max_ = 539 nm; beta-λ_max_ = 348 nm; *a*_0_,_1_ for alpha band: 390, 9.9, respectively; *a*_0_,_1_ for beta band: 211, 6.4. See Materials and Methods for values tabulated in [15].

Spectral sensitivity was measured by presenting 10 ms flashes at various wavelengths, using neutral density filters to adjust flash intensity so that the range of response amplitudes were along the middle, approximately linear, region of the *V*/log*I* function. For green photoreceptors, peak spectral sensitivity was at 541.1±8.5 nm (range 530–554; n = 9), estimated by fitting equation 2 [15] to calculated sensitivity values at 500–600 nm for each cell. Normalized averaged data for all green photoreceptors (Fig. 2A) could be well-fitted by the modified template of Stavenga et al. [15] (equations 3,4) by allowing the shape parameters to vary from the tabulated values (Fig. 3). This yields an estimate for the peak spectral sensitivity of 539 nm, with a secondary (beta) peak in the UV at 348 nm.

Using a 6^th^ degree polynomial, as described by Baylor et al [16], the data could be equally well fit (r^2^ = 0.994 in either fit), yielding a peak of 537.8 nm.

Spectral sensitivity peaks for blue photoreceptors ranged from 417–431 nm (mean: 424.1±5.0, n = 5). Despite the fairly small number of cells contained in this sample, this result indicates that the λ_max_ of the *Bombus impatiens* blue photoreceptor is at a significantly shorter wavelength than that of *Bombus terrestris*, measured with identical techniques (437.0±2.7, n = 18; p = 0.0033) [11]. Normalized, averaged data for all 5 *Bombus impatiens* blue photoreceptors are plotted in Fig. 2B.

Spectral sensitivity peaks for UV photoreceptors ranged from 341–349 nm (mean: 345.8±6.4, n = 6). The best fit to the normalized averaged data gave a spectral peak of 347.3 nm (Fig. 2C).

## Discussion

Our results indicate that the *Bombus impatiens* worker bee has trichromatic colour vision, with photoreceptor spectral sensitivity peaks for UV, blue and green photoreceptors well within the ranges reported for other hymenopterans. In a previous study of spectral sensitivity in *Bombus terrestris* [11] we compared photoreceptor spectral sensitivities between two subspecies: *Bombus terrestris dalmatinus* (Southern European mainland) and *Bombus terrestris sassaricus* (native to Sardinia). We found a small but significant difference in the λ_max_ estimates for the green photoreceptor between the two populations: 533 and 538 nm, respectively. Our estimated λ_max_ from averaged spectral data from *Bombus impatiens* green photoreceptors was 539 nm, which is closer to that for the Sardinian population; however the spread in our data was such that there was no statistically significant difference with either of the *Bombus terrestris* subspecies. Peak sensitivity estimates for *Bombus terrestris* UV (347–348 nm) and blue photoreceptors (435–436 nm) did not differ between subspecies [11]. In the present study we find a similar value for the UV receptor λ_max_ (346 nm) but a significantly short wavelength shifted peak for the blue receptor (424 nm). Equivalent values for 8 other species of Apidae in the study of Peitsch et al. [10] range from 328–352 nm for UV photoreceptors, 428–440 nm for blue photoreceptors, and 524–548 for green photoreceptors.

Our electrophyiologically-measured spectral sensitivity data deviates significantly from the theoretical absorbance curves calculated for vitamin A1-based visual pigments, most notably in the lower sensitivity values for the secondary, UV peaks of the blue and green photoreceptors (corresponding to the beta absorbance band), and also in the narrower bandwidth of the main peaks of the UV and blue photoreceptors. The electrical signal generated by a photoreceptor depends primarily on its photopigment, but clearly there can be many other interacting factors, especially in the *in situ* recordings used here, where the optics of the eye are essentially intact. Such considerations can include self-screening and wave-guide effects [17] as well as the presence of screening pigments [18].

The theoretical basis of most attempts to derive universal photopigment spectral sensitivity templates is that the form of the resulting curve is shape invariant when plotted on the *λ*/*λ*_max_ axis; in this way the shape of the spectral absorbance curve is uniquely defined by *λ*_max_ [15], [19], [20]. However, although the main peak of the green photoreceptor can be fairly well described by the template of Stavenga et al. [15], as well as others such as the equation of Lamb [19] as modified by Govardovskii et al. [20], the bandwidths of the main peaks of the blue and UV photoreceptors are significantly narrower (this is especially true for the blue photoreceptor, where interaction with the beta absorbance band would be expected to lead to considerable broadening of the main peak). As discussed above, electrophysiological measurements may only indirectly reflect underlying photopigment absorbance spectra, but we also note that there can be deviations from predicted spectra even when based on isolated photoreceptor photocurrents: for example recordings from amphibian cones have revealed narrower than predicted spectra [21].

On the basis of their central projections, insect photoreceptors fall into two classes: short visual fibres, which project to the lamina (the first optic neuropile), and long visual fibres, which project directly to the medulla (the second optic neuropile) [22]. In the honeybee each ommatidium contains six green photoreceptors, which give rise to the short visual fibres. The remaining two principal photoreceptors (retinula cells R1 and R5) give rise to long visual fibres. These cells can be either blue or UV photoreceptors, giving rise to an eye with three types of ommatidia: those containing two blue photoreceptors, two UV photoreceptors, or one blue and one UV photoreceptor [23]. In *Bombus impatiens* a similar pattern is likely, since UV opsin is found in zero, one or two receptors per ommatidium, and these cells always correspond to the R1 or R5 photoreceptors [24]. It seems likely, therefore, that the green photoreceptors identified here correspond to the short visual fibres, and the blue and UV photoreceptors to the long visual fibres.
